# Supplementary material for: Characterization of New ATM Deletion Associated with Hereditary Breast Cancer
Source: Genes (Basel). 2021 Jan 21;12(2):136. doi: 10.3390/genes12020136 (PMC7911716; doi:10.3390/genes12020136)
Supplement: Supplementary file 1 [file genes-12-00136-s001.pdf]

P041-ATM kit 3244 negative ctrl

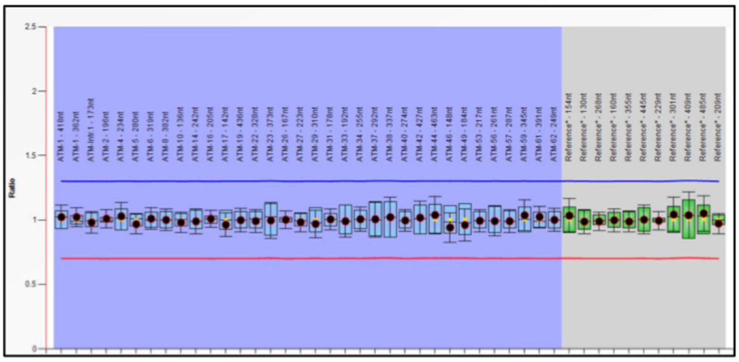

P042-ATM kit 3244 negative ctrl

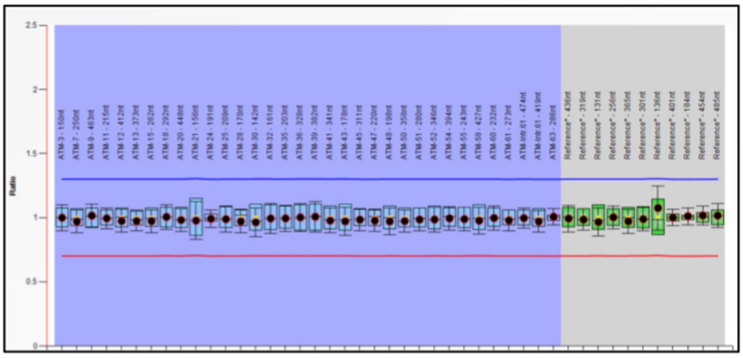

P041-ATM kit 3167 proband

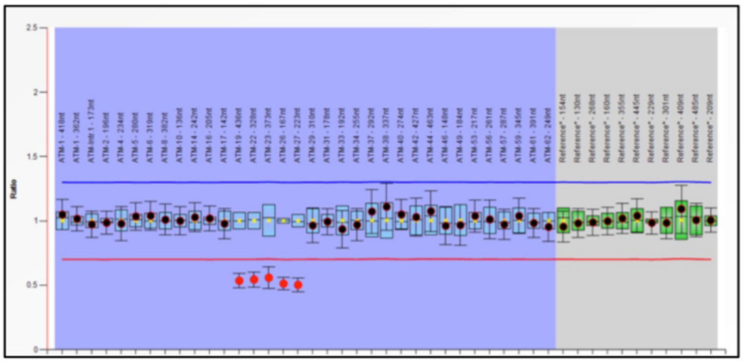

P042-ATM kit 3167 proband

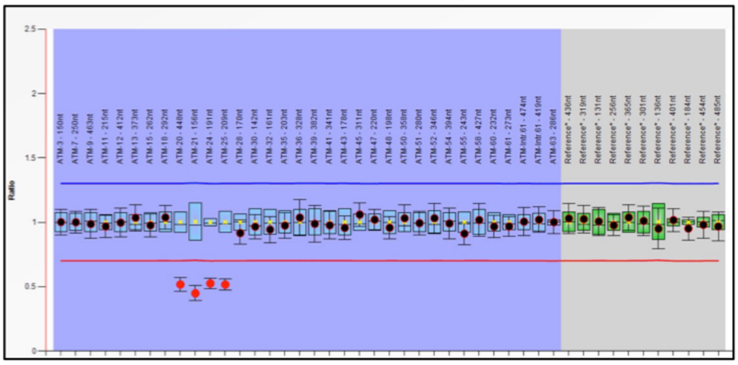

Supplementary Fig, 1
